# Feasibility of Spectral Analysis as a Tool in Nursing Research to Quantify Patterns of Respiration in Premature Infants

**DOI:** 10.1155/2024/6671906

**Published:** 2024-01-27

**Authors:** Khlood Bubshait, Olivia Dizon, Charlene Krueger

**Affiliations:** ^1^Fundamental of Nursing Department, Imam Abdulrahman Bin Faisal University, Dammam 1980, Saudi Arabia; ^2^University of Florida, Gainesville, FL 32610-0197, USA

## Abstract

**Background:**

Respiratory difficulties are a common concern in preterm infants, and they can lead to long-term health problems. Few studies have investigated the use of spectral analysis as a biomarker to quantify respiration patterns in preterm infants.

**Objective:**

To evaluate the feasibility of using spectral analysis of heart rate variability as a biomarker for the quantification of respiratory patterns in very-low-birth-weight preterm infants compared to direct observation.

**Methods:**

In a comparative, small-scale feasibility study, 18 preterm infants born during their 27^th^ to 28^th^ gestational week (weighing <1500 grams) participated by convenience. Respiratory patterns (regular or irregular; shallow or deep) were directly observed on the 28^th^ week during playback of speech recording. Heart rate variability was simultaneously measured using spectral analysis of heart periods, from which the mean values influenced by respiratory sinus arrhythmia (frequencies of 0.30–1.0 Hz) were compared to each observed respiratory pattern. The magnitudes of respiratory sinus arrhythmia and the area under the curve were determined.

**Results:**

The magnitude of respiratory sinus arrhythmia (frequencies of 0.30–1.0 Hz) in infants observed to be displaying irregular shallow respiration was greater than that in infants with regular deep respiration. Further, there was a shift from lower frequencies (frequency peak = 0.30 Hz) to higher frequencies (peak = 0.70 Hz).

**Conclusion:**

In contrast with direct observation, spectral analysis allowed for the quantification of respiratory patterns in a vulnerable population of preterm infants of interest to the nursing scientific and practice community. Future directions include applying this biomarker to evaluate both developmental and pathological trends in the respiratory patterns of preterm infants.

## 1. Introduction

Breathing patterns in preterm infants consist of extremely variable interbreather intervals that are hypothesized to originate from the respiratory oscillator and its input-output responses to central and peripheral signals [1]. Common patterns include shallow, irregular, deep, or regular breathing patterns, and previous studies have revealed that respiratory apnea can be associated with patterns or periodicities in breathing-over-time scales [2]. Continuous monitoring of respiratory patterns for preterm infants is therefore critical to early detection and treatment [3, 4].

Spectral analysis of heart rate variability (HRV) as a complementary measure of respiratory patterns is relevant to preterm infants due to its ability to substantiate the maturation of the autonomic nervous system (ANS) in infants [5–7]. Direct observation (both visually and direct monitoring) is currently used in neonatal intensive care units (NICUs) to assess respiratory patterns in preterm infants. Typically, the reliability of direct observation in research is assessed using either intra- or interobserver agreement. However, analogues to internal consistency for interobserver agreement are not always consistent with interobserver agreement [8–11]. The addition of spectral analysis of HRV as a tool for respiratory patterns, however, has the potential to allow for reliable quantification of different types of respiration in preterm infants. The purpose of this study, therefore, was to evaluate the feasibility of using spectral analysis of HRV to quantify patterns of respirations in very-low-birth-weight (VLBW) preterm infants compared to direct observation.

### 1.1. Preliminary Work on Maternal Voice

#### 1.1.1. Heart Rate Variability in Preterm Infants (NIH/NINR P20 NR07791 and NIH/NCRR MO1 RR00082)

This data was obtained during 300-second silent period (when no voice is played back). The high-frequency components of group (*F* = 5.16; df 1/46; *p* = 0.0278), gender (*F* = 4.88; df 1/46; *p* = 0.0323), and time^∗^ gender interaction (*F* = 6.59; df 1/46; *p* = 0.0136) were found. Overall, high-frequency components were greater for infants' hearing for 7 weeks (group 1) compared to those hearing for only 2 weeks (group 2) (group 1: 21.78, SE = 2.36; group 2: 21.65, SE = 2.20) and for females compared to males (males = 19.07, SE = 2.47; females = 23.29, SE = 3.02). A significant interaction between time and gender was shown, in that females tended to begin the study with lower levels of high-frequency components compared to males (males = 17.09, SE = 1.62; females = 14.12, SE = 0.72) but finished higher at study completion (males = 17.20, SE = 2.36; females = 31.61, SE = 2.27) [12].

## 2. Materials and Methods

### 2.1. Study Design

A comparative, small-scale feasibility study was employed to complete a secondary analysis of data involving 18 VLBW infants (weighing <1500 grams) born during their 28th-week postgestational week and their mothers. This study is a part of the primary study which was conducted using a randomized clinical trial with a longitudinal and repeated-measure design on 49 preterm infants.

### 2.2. Recruitment

**Figure 1:**
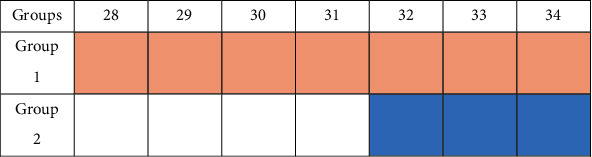
Sample assignment.

This study was approved by the University of Florida Institutional Review (NIH/NINR P20 NR07791 and NIH/NCRR MO1 RR00082). Informed consent was obtained from the participant mothers for themselves and their infants. Potential subjects were identified via frequent review of the admission log for NICU. The research team accessed the name and gestational age of the infants in order to determine if the infant potentially met inclusion criteria for enrollment. No record was made of these identifiers, but this information was used to initiate potential enrollment. Contact with the mother for enrollment of herself and infant was begun by first asking the primary nurse whether the mother was interested and was approachable for potential participation in the study. If the mother indicated an interest in study participation, the nurse contacted the research team to begin the consent process. Mothers consented at the bedside of their infant and or/within a private room within the hospital. In order to minimize the possibility of coercing or unduly influencing subjects, the consent form was read and presented by the researchers, and mothers were not approached for enrollment until 24 hours following the birth of their preterm infant. After mothers signed the informed consent forms, infants were randomized to 1 of 2 groups by a computer-generated system. Infants were randomly assigned to either group 1 (exposed daily to maternal speech recording from 28 to 34 weeks' PMA) or group 2 (exposed daily to maternal speech recording from 32 to 34 weeks' PMA) as shown in Figure 1. The exposure dose (the mean number of minutes the infant heard the maternal recording) for group 1 is 6 weeks, twice daily, while for group 2 is 2 weeks, twice daily. Measurements of HRV during playback of the familiarized rhythm recited by a stranger's speech occurred weekly for 7 weeks in both groups.

For this study, we selected 18 participants as they have complete data during the playback recording period, and we want to test the feasibility.

### 2.3. Setting and Target Population

The study was conducted while the infants were cared for in a level III NICU in the southeast United States, a primary referral center. Preterm infants with their mothers (*n* = 18) were sampled by convenience for the study.

Maternal and infant inclusion criteria were as follows: (1) birth occurring during the 27^th^ to 28^th^ gestational week (to control for maturational effects on outcomes), (2) birth weight < 1500 grams (very low birth weight), (3) infants' mothers between 18 and 35 years of age (for legal participation in research and to avoid age-related risk factors), and (4) mothers who underwent a first- or second-trimester ultrasound (to confirm the gestational age of the fetus during the pregnancy).

Maternal and infant exclusion criteria were as follows: (1) an abnormal head ultrasound, (2) sensorineural hearing loss, (3) a confirmed prenatally transmitted viral/bacterial infection, and (4) cardiac abnormalities.

### 2.4. Procedure

A mother's speech is both a predominant and unique source of early sensory stimulation for the developing fetus and thus a logical focus for researchers interested in the development of interventions that use maternal speech as an auditory source of sensory stimulation for preterm infants. While other sources of sound are largely unimodal for the developing fetus (heartbeat and bowel sounds), maternal speech is multimodal and provides a combination of auditory, vibratory, and vestibular stimulation for the developing fetus.

In human premature infants, the exposure usually occurs from high sound levels of different external environmental stimuli in the NICU such as the cardiac monitor, health care providers' conversation, parents' talk, and pagers. These stimuli usually have a sound level of more than 55 decibels (dB) which is above the hearing level of premature infants. Some preliminary evidence revealed that these stimuli possibly affect the level of neurobehavioral development in premature infants. According to Krueger, the mother's voice along with other sensory stimuli are vital in improving and shaping the neurobehavioral development in normal premature infants. Therefore, this study investigated the effect of early developmental exposure to mothers' voice on respiratory patterns in premature infants.

All preterm infants enrolled in the study were tracked weekly from 28 to 34 weeks' postmenstrual age (PMA). Study procedures (maternal-controlled exposure and testing sessions) were performed while preterm infants remained in their assigned incubator beds.

### 2.5. Auditory Stimuli

One nursery rhyme was used in this study and recorded by the mother (maternal recording) and an unfamiliar female (test recording). The untitled rhyme was nine lines long, took approximately 15 seconds to recite, and was not a common verse, making it unlikely that the infants would be spontaneously exposed to it. Both maternal and test recordings of the rhyme were made by asking the speaker to use “motherese.” *Motherese* is a method of speech that emphasizes greater pitch changes and slower speech. The recordings lasted approximately 45 s, consisting of three repetitions of the rhyme and were calibrated using Adobe Audition software to preset sound levels to 55 ± 5 dB and remove all low-frequency noise [8]. This level was chosen in order to maintain the decibel level just below the normal level of human speech (58-60 dB) and to remain within recommended sound levels for the preterm infant.

### 2.6. Neonatal Monitor

Heart rate and heart rate variability were obtained from a series of heart periods recorded from each infant's electrocardiogram (EKG) signal. Heart periods were sampled at a rate of 500 Hz during two epochs of time: (1) a 300 s silent epoch and (2) a 45 s epoch during which the recording of the unfamiliar female was played back to the infants. The EKG signal was then transferred to a Dell Inspiron 8100 laptop, using EKG electrodes attached in the standard three-lead manner via an RS232 interface and software from an Agilent neonatal monitor (model #1092A). A MATLAB program was used to filter or smooth the signal by removing noise components and baseline wander. The filtered signal was next passed through a QRS detection algorithm (also implemented in MATLAB), resulting in a time series of R-R intervals or heart periods.

### 2.7. Maternal-Controlled Exposure

Controlled exposure to the maternal recording varied depending upon group assignment. Maternal recordings were played back twice daily at 10 am and 2 pm. Group 1 infants heard twice daily from 28 to 34 weeks' PMA, and group 2 infants waited four weeks and heard twice daily from 32 to 34 weeks' PMA. A control group was therefore not utilized in the current study. Once approached for study participation, the authors believed that mothers would be reluctant to participate if their infant could potentially be randomly assigned to not hear a recording of their voice reciting the nursery rhyme. As an alternative, we chose to stagger the groups, allowing for group 2 to essentially function as a control group up until the 32nd-week PMA test session.

Recordings were broadcast using a speaker (Pillowsonic) positioned within the infants' cribs via small MP3 players programed to play twice a day. Mothers and healthcare providers were blinded to group assignment. The playback system was set up for all infants (independent of group assignment) during the first week of participation in the study or at 28 weeks' PMA.

Monitoring of maternal-controlled exposure consisted of an initial calibration of the decibel levels using the A-scale of a Bruel-Kjaer (220SLM) sound level meter for playback at 55 dB ± 5 db. This was followed by visits of a research team member to the infants' bedsides to check the playback system for any interruptions in the playing of recordings related to bed changes, items placed on top of the speaker, and/or misplacement of the speaker.

### 2.8. Weekly Test Sessions

Test sessions were conducted weekly and only initiated if the bedside nurse deemed the infant stable. With each test session, data collection was begun at 45 s before, 45 s during, and 45 s after the stimulus epoch in which the recording of the unfamiliar female was played back to the infant. The recording of the unfamiliar female was played back using a pillow speaker (similar to that used for maternal-controlled exposure) approximately 10 cm from the infant's head. Playback of the recording was not initiated until the infant was determined to be in an active sleep state. Criteria established for use with preterm infants were employed to detect an active sleep state. The subject was determined to be in an active sleep state if (1) the respirations were irregular, (2) no gross motor body movement was present, (3) eyes were closed, and (4) heart rate variability < 10 beats per minute (performed post hoc). The EKG signal was then transferred to a Dell Inspiron 8100 laptop, using EKG electrodes.

### 2.9. Direct Observation

A direct visual observation of respiratory patterns for 45 seconds during playback of the recording was performed by two researchers via their eyes without any equipment.

Direct observation was done by using the bare eye (sense of vision) to observe the movement of the infant's thorax and determine the respiratory pattern.

A spectral analysis of HRV was measured using EKG to determine the values of RSA.

### 2.10. Outcomes

Direct observation of the respiratory pattern and spectral analysis of HRV were measured simultaneously during one 45 s epoch of time.

#### 2.10.1. Direct Observation

The respiratory patterns (regular or irregular; shallow or deep) were directly observed with >95% interrater reliability.

#### 2.10.2. Spectral Analysis

HRV was obtained from a series of heart periods recorded from each infant's EKG signal during the test session. Heart periods were sampled at a rate of 500 Hz. The EKG signal was then transferred to a Dell Inspiron 8100 laptop, using EKG electrodes attached in the standard three-lead manner via an RS232 interface and software from an Agilent neonatal monitor (model #1092A). A MATLAB program was used to filter or smooth the signal by removing noise components and baseline wander. The filtered signal was next passed through a QRS detection algorithm (also implemented in MATLAB), resulting in a time series of R-R intervals or heart periods. Thus, HRV was measured using spectral analysis of heart periods and evaluated using the magnitude of the mean respiratory sinus arrhythmia (RSA) value and area under the curve (AUC).

### 2.11. Statistical Analysis

Data were assessed and analyzed using SAS software, version 9.2 (SAS Institute, Inc., Cary, NC). Comparisons between direct observation and HRV were then made based on the respiratory pattern directly observed during the test session. Infants were assigned to one of two groups post hoc: group 1 (direct observation of regular deep respirations) and group 2 (direct observation of irregular shallow respirations). Next, the magnitudes of RSA and AUC for the respiratory sinus arrhythmia were calculated for both group 1 and group 2.

## 3. Results and Discussion

### 3.1. Demographic Characteristics

The total sample size for this study was 18 infants. There was no difference in the baseline demographics and clinical characteristics of the mothers and their infants between the two groups by maternal age, ethnicity, delivery route, infant gender, Apgar score, and birth weight. A description of the study population is given in Figure 1.

The mothers' mean age in group 1 was 22.69 (SD = 6.35) whereas in group 2 was 26.71 (SD = 6.89). While there was no difference by delivery route between groups, clinical differences were noted. The percentage of delivery route between groups 1 and 2 (group 1: vaginal route = 44% and cesarean section = 56%; group 2: vaginal = 29.17% and cesarean section = 70.83%). A description of the study population is given in Table 1.

**Figure 2:**
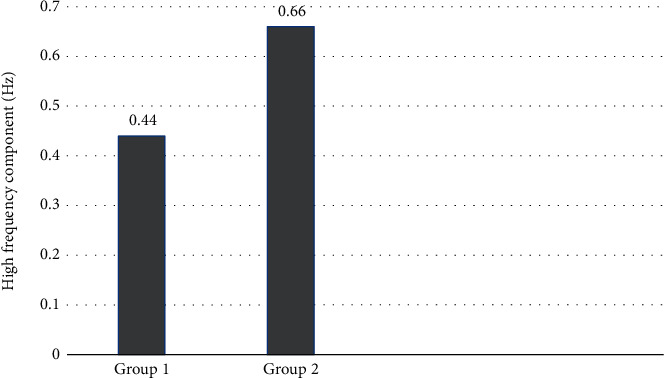
The magnitude of mean RSA values (frequencies 0.30–1.0 Hz) in group 2 infants (displaying irregular shallow respirations) is greater than that in group 1 infants (displaying regular deep respirations).

**Figure 3:**
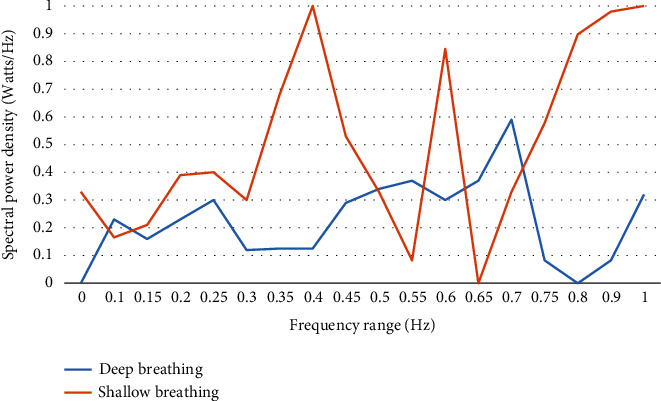
High-frequency curve (0.3 to 1.00 Hz) of deep breathing versus shallow breathing at 34 weeks' PMA.

In this study, we evaluated whether spectral analysis of HRV is a feasible biomarker in quantifying respiratory patterns in preterm infants. The analysis highlighted the differences between direct observation and HRV measures in quantifying respiratory patterns based on the magnitude of mean RSA values and the peak within the AUC. An increase in the magnitude of RSA was greater in infants with irregular shallow respirations than in infants with regular deep respirations as shown in Figure 2. However, breathing rates varied significantly both within and between the infants. Further, as shown in Figure 3, the AUC was sensitive to RSA and displayed a sharp shift to higher-frequency power (peak = 0.70). The interrater reliability of each variable is reported separately. For each variable, the raw values between the two raters were compared (the score created for a numerical value was assigned to patient characteristics). This was done using a formula in SPSS, eliminating the possibility of error in calculating the score. Reliability of the respiratory patterns' observation score was assessed with a weighted kappa.

The RSA variations suggest vagal phasic pattern differences in the heart rate [13]. Two significant areas of caution are as follows: (1) breathing indicators may affect the relationship between RSA and the parasympathetic nervous system and (2) the RSA amplitude is influenced by sympathetic modulation. In addition, the RSA is considered a stress-linked reactivity and depends on the types of stressors the infants are exposed to. The RSA magnitude that decreases to stimulus indicates a vagal withdrawal while the RSA magnitude that increases to stimulus indicates a proper cortical-subcortical functioning (efficient cognitive managing of emotional stimuli) [14]. Taken together, these issues should be considered in the accurate interpretation of the RSA as a marker of parasympathetic nervous system regulation of the heart rate. The limitations of this feasibility study include the small sample size, use of a nonrandom sampling method, use of a nonlongitudinal design to compare between and within subjects' effects, and variations in nursing routine care and feeding which potentially interfered with obtaining an accurate RSA measurement. Future studies may include longitudinal quantification of respirations over multiple gestational weeks and a random sampling investigation between and within subjects' effects by using an appropriate statistical model. In addition, demographic variations may be studied, comparing between males and females, using different gestational ages, and applying this potential biomarker to evaluate both developmental and pathological trends in the respiratory patterns of preterm infants. In contrast with direct observation, spectral analysis of heart periods allowed for quantification of respiratory patterns in a vulnerable population of preterm infants of interest to the nursing scientific and practice community. In neonatal clinical settings, monitoring the respiratory patterns of preterm infants allows for easier recognition of physiological instability; thus, it can provide valuable insights into the cardiorespiratory status of the neonate, as well as their maturational stage.

## 4. Conclusion

The results of this study suggested that quantifying respiratory patterns by using spectral analysis of HRV is feasible in preterm infants and warrants additional research in this vulnerable population.

## Figures and Tables

**Table 1:** Demographic data of mothers and their preterm infants.

Variables	Overall (*n* = 49)	Group 1 (*n* = 24)	Group 2 (*n* = 25)
Mother's age (year)	24.80 (6.35)	22.69 (5.29)	26.71 (6.89)
Mother's ethnicity			
White	67.35%	68%	66.67%
African American	30.61%	32%	29.17%
Hispanic	2.04%	00	70.83%
Delivery route			
Vaginal	36.73%	44%	29.17%
C-section	63.27%	56%	70.83%
Infants' gender			
Male	40.81%	20.40%	20.40%
Female	59.18%	30.61%	28.57%
Infants' birth weight (gram)	1080 (193.20)	1100.16 (120.30)	1058.30 (251.00)
Infants' Apgar score			
1 minute	5.65 (2.28)	5.84 (2.08)	5.43 (2.52)
5 minutes	7.17 (1.60)	7.08 (1.78)	7.26 (1.42)

Note: value represents the mean and standard deviation of maternal and infants' characteristics.

## Data Availability

The original data used to support the findings of this study is available from the corresponding author upon requested.
